# Global health trends of human papillomavirus‐associated oral cancer: A review

**DOI:** 10.1002/puh2.126

**Published:** 2023-10-03

**Authors:** Kehinde Kazeem Kanmodi, Jimoh Amzat, Jacob Njideka Nwafor, Lawrence Achilles Nnyanzi

**Affiliations:** ^1^ Faculty of Dentistry University of Puthisastra Phnom Penh Cambodia; ^2^ School of Dentistry University of Rwanda Kigali Rwanda; ^3^ Campaign for Head and Neck Cancer Education (CHANCE) Programme Cephas Health Research Initiative Inc Ibadan Nigeria; ^4^ School of Health and Life Sciences Teesside University Middlesbrough UK; ^5^ Department of Sociology Usmanu Danfodiyo University Sokoto Nigeria; ^6^ Department of Sociology University of Johannesburg Johannesburg South Africa; ^7^ Division of Medicine Nottingham University Hospitals NHS Trust Nottingham UK; ^8^ Faculty of Public Health King Ceasar University Kampala Uganda

**Keywords:** challenges, control, global health, human papillomavirus, oral cancer, recommendations

## Abstract

Oral cancer is a malignant neoplastic disease, it constitutes the most common cancer in the head‐and‐neck region, and it is the 16th most common and 15th deadliest cancer type affecting humans. Despite the global efforts channelled towards global cancer control, the burden of oral cancer is still increasing. Every year, several thousands of people across 195 countries/territories die of oral cancer, and only about 40%–50% of the victims of oral cancer barely survive for 5 years after diagnosis. Within the past few decades, there has been a paradigm shift in the epidemiological trend of oral cancer risk factors. Oral sex – a major route of transmission of human papillomavirus (HPV) – was found to be increasing at an alarming rate, whereas tobacco and alcohol use had been on the decline. This narrative review critically discussed the contemporary challenges facing the global eradication of HPV‐associated oral cancer, with reasonable recommendations. The challenges discussed in this review are major, ranging from clinical to public health policy issues, which include the lack of validated and inexpensive oral HPV screening and diagnostic tools, high global public illiteracy on oral HPV infection, diverse sociocultural factors, weak global research capacity on oral HPV infection and HPV‐associated oral cancer and the absence of policies across different countries on national preventive programmes on HPV‐associated oral cancer. This review also identified the need for more attention towards the prevention and control oral HPV infection and HPV‐associated oral cancers globally.

## INTRODUCTION

Oral cancer, a malignant neoplasm of the oral cavity and oropharynx, is a disease which has afflicted humans for over three millennia [1, 2, 3, 4, 5]. Moreover, it is the most common cancer in the head‐and‐neck region, and it is the 16th most common and 15th deadliest cancer type affecting humans [6, 7, 8, 9, 10]. Cases and mortalities of oral cancer have been reported in all genders, races, regions, countries and virtually all age groups [9, 10, 11, 12]. Every year, several thousands of people across 195 countries/territories die of oral cancer, and only about 40%–50% of oral cancer victims barely survive for 5 years after diagnosis [9, 13]. In 2020 alone, about 476,125 newly diagnosed oral cancer cases and 225,900 new oral cancer‐associated deaths were recorded globally [14]. Despite the global efforts channelled towards global cancer eradication, the burden of oral cancer is unabating. This demonstrates that oral cancer is a serious issue of global health concern.

The current economic burden of oral cancer is so enormous. In 2017 alone, the disability‐adjusted life‐years of oral cancer was 64.23 years per 100,000 persons [9]. By interpretation, this means that for every 1000 persons in the year 2017, 0.64 year were lived in disability and/or were lost prematurely due to oral cancer [9, 15]. Furthermore, the cost of care of oral cancer is very expensive even at the early stages of its diagnosis [16]. For example, in the Western world, the per capita costs of oral cancer care range from 18% to 127% [16]. It is well known that the populations in Western countries are generally richer than those in non‐Western countries; hence, the economic impacts of oral cancer care costs will be more devastating in several non‐Western countries due to deep‐seated poverty and limited access to healthcare among those populations [17, 18, 19].

In recent peer‐reviewed reports on oral cancer, it was reported that the incidence and prevalence rates of human papillomavirus (HPV)‐associated oral cancers have significantly increased over the years, increasing the overall global health burden of the disease [14, 20, 21, 22]. Current findings revealed that at least 18%–28% of global oral cancer cases are attributed to HPV [14, 23], and this is because HPV is one of the commonest sexually transmitted viruses among humans [5, 20, 21, 22, 24]. Overall, this shows that HPV‐associated oral cancer is a serious global public health problem.

HPV infection is highly preventable through vaccination [25]. Unfortunately, the global coverage of the vaccine is very limited as several countries, especially the low‐ and middle‐income countries (LMICs) are yet to incorporate HPV vaccination into their national routine immunisation programmes [26]. Moreover, there are other major factors that have persistently precipitated HPV infections globally, all of which are cumulatively contributed to the rising global prevalence of oral cancer. To stem the rising global scourge of oral cancer, it is imperative to understand the contemporary challenges associated with HPV‐associated oral cancer eradication.

This narrative review aimed to critically review the global epidemiological trajectory of oral cancer burden and its risk factors – with a focus on HPV, to identify the contemporary challenges associated with global oral HPV infection eradication and to provide insights towards strategies that can be adopted to curb the global burden of HPV‐associated oral cancers.

## METHODS

The literature used in this narrative review were obtained from online search of multiple electronic databases – CINAHL, AJOL, PubMed, SCOPUS, PsycINFO, Web of Science, EBSCO, Embase and Google Scholar – with the aid of Boolean operators and multiple search terms, including ‘oral cancer’, ‘oral squamous cell carcinoma’, ‘oral malignancy’, ‘oral neoplasia’, ‘human papillomavirus’, ‘HPV’, ‘global health’, ‘tobacco’, ‘alcohol’, ‘policy’, ‘screening’, ‘control’, ‘prevention’, ‘trends’, ‘public health’, ‘international health’, ‘global health’, and ‘epidemiology’. The searches were done between 01 October 2021 and 01 December 2022, and from the search, relevant articles, reviews, datasets, books, book chapters, policy documents and grey literature, including website‐based information which was resourceful for this review, were obtained. The data obtained from these sources informed the discourse of the review.

## EPIDEMIOLOGY OF HPV‐ASSOCIATED ORAL CANCER

Based on the 2017 Global Burden of Disease study, the global burden of oral cancer is enormous [9, 10]. The age‐standardised incidence rate (ASIR) of oral cancer is 4.84 cases per 100,000 people [9, 10] (Table 1). However, at the regional level, South Asia (10.71 per 100,000) was the region with the highest ASIR of oral cancer, whereas the North Africa and Middle East region (1.48 per 100,000) had the lowest oral cancer ASIR [9]. At the country level, the ASIR of oral cancer was highest in countries with low‐middle socio‐demographic index (SDI) (7.45 cases per 100,000), and lowest in countries with high‐middle SDI (3.14 cases per 100,000) (Table 1).

**TABLE 1:** Estimated age‐standardised incidence rates and age‐standardised mortality rates of oral cancer for the year 2017.

Geographical location	Estimated 2017 ASIR (per 100,000 persons)	Estimated 2017 ASMR (per 100,000 persons)
Worldwide	4.84	2.42
Per socio‐demographic index (SDI):		
High SDI	5.00	1.50
High‐middle SDI	3.14	1.51
Middle SDI	4.20	2.26
Low‐middle SDI	7.45	4.70
Low SDI	5.94	4.34
Per region:		
South Asia	10.71	6.95
Australasia	5.91	1.68
High‐income North America	5.59	1.30
Eastern Europe	4.96	2.51
Western Europe	4.90	1.53
Central Europe	4.74	2.70
Southeast Asia	4.65	2.44
Oceania	4.35	2.68
Caribbean	4.09	2.29
Tropical Latin America	3.96	2.27
Southern Sub‐Saharan Africa	3.68	2.44
High‐income Asia Pacific	3.50	1.10
Eastern Sub‐Saharan Africa	2.97	2.23
Central Asia	2.96	1.73
Southern Latin America	2.72	1.28
East Asia	2.71	1.17
Central Sub‐Saharan Africa	2.69	2.06
Andean Latin America	1.96	1.07
Central Latin America	1.89	1.01
Western Sub‐Saharan Africa	1.85	1.31
North Africa and Middle East	1.48	0.78

*Source*: Ref. [9].

The global age‐standardised mortality rate (ASMR) of oral cancer was 2.42 cases per 100,000 people [9] (Table 1). At the regional level, South Asia (6.95 per 100,000) had the highest oral cancer ASMR, whereas the North Africa and Middle East region (1.5 per 100,000) had the lowest oral cancer ASMR [9] (Table 1). At the country level, oral cancer ASMR was highest in countries with low‐middle SDI (4.70 per 100,000) but lowest in countries with high SDI (1.50 per 100,000) (Table 1).

Within the last three decades (1990–2017), the epidemiological burden of oral cancer had worsened [9]. A comparative analysis of the global epidemiological data on OC indicates that the global ASIR and ASMR of oral cancer increased by onefold between 1990 and 2017 (an 18‐year‐interval) (ASIR – 109.6%; ASMR – 98.7%) [9] (Table 1). From the review of multiple literature, it can be asserted that the rise in the incidence of HPV‐associated oral cancers largely contributed to the overall increase in the global epidemiological burden of oral cancers [14, 20, 21, 22, 23, 27] (Table 2). Currently, at least 18%–28% of the global oral cancer cases are attributed to HPV [14, 23]. This further confirms that HPV‐associated oral cancer is an issue of global health concern.

**TABLE 2:** Some examples of change in the epidemiological trend of human papillomavirus (HPV)‐associated oral cancers.

Country/population	Period	Oral cancer site	Notable findings
Taiwan [28]	1995–2009	Tonsil	The annual percentage change in the incidence rate of HPV‐positive tonsillar cancer was 8.2%
Taiwan [28]	1995–2009	Base of the tongue and lingual tonsil	The annual percentage change in the incidence rate of HPV‐positive cancer of the base of the tongue and lingual tonsil was 5.6%
Sweden [29]	1998–2007	Base of the tongue	There prevalence of HPV‐positive base of tongue cancer has increased over the years (from 58% in 1998–2001 to 84% in 2006–2007)
United States [30]	2010–2015	Oropharynx	The prevalence of HPV‐positive oropharyngeal cancers has significantly increased over time at a rate of 3.5% and 3.2% per year among men and women, respectively
Germany [31]	2000–2017	Oropharynx	An annual change of +1.015 was reported in the yearly prevalence of HPV‐positive oropharyngeal cancers
Denmark [32]	2000–2017	Oropharynx	The annual prevalence of HPV‐positive oropharyngeal cancers has increased by threefold
Brazil [33]	2017–2019	Oropharynx	The prevalence of HPV‐positive oropharyngeal cancers was 21.1%, 31.6% and 32.4% in 2017, 2018 and 2019, respectively
Italy [34]	2010–2019	Oropharynx	There was a significant increase overtime in the rate of HPV‐positive oropharyngeal cancer cases (40.3% in 2010–2014 vs. 53.7% in 2015–2019, *p‐value* = 0.019)

## MAJOR ORAL CANCER RISK FACTORS

Oral cancer has diverse risk factors, and they include tobacco use [35, 36, 37], betel (areca) nut chewing [35], alcohol consumption [36], chronic occupational exposure to some petroleum‐based and oxygenated solvents (e.g. tetrahydrofuran, diethyl ether and benzene) [38], viral infections (by human immunodeficiency virus (HIV), Epstein Barr virus, HPV) [35, 39, 40, 41], ultraviolet radiation [35, 42] and undernutrition [43, 44, 45, 46]. Of the diverse oral cancer risk factors, tobacco use, alcohol use and oral sex (a transmission route for HPV infection) are the three major risk factors of oral cancer globally [47, 48].

Over the years, there has been a significant decline in the prevalence trends of the global burden of tobacco use [49]. According to the most recent WHO report [49], the global prevalence of tobacco consumption amongst adults (people aged 15 years and above) decreased from 33.3% in 2000 to 24.9% in 2015; also, further decline was projected to occur in coming years (Table 3). This shows that tobacco use continues to be a diminishing threat to the global oral cancer burden.

**TABLE 3:** Global age‐standardised prevalence trend of tobacco smoking and alcohol use among adults aged 15 years and above.

Year	Global age‐standardised prevalence (%) of tobacco smoking
2000	33.3
2005	30.1
2010	27.3
2015	24.9
2020	22.8
2025 (projected)	20.9
**Year**	**Global prevalence (%) of heavy episodic alcohol drinking**
2000	22.6
2005	20.5
2010	20.5
2016	18.2
**Year**	**Global prevalence (%) of total alcohol consumption per capita**
2016	43.0
2020	41.7
2025 (projected)	40.3

*Source*: Refs. [49, 50].

The global prevalence rate of alcohol consumption (including heavy episodic alcohol drinking) has decreased over the last decade [50]. According to the most recent WHO report [50] on alcohol use burden, the global prevalences of heavy episodic alcohol drinking per capita among people aged 15 years and above have decreased from 22.6% in 2000 to 18.2% in 2016; furthermore, the total global alcohol consumption per capita has decreased from 43.0% in 2016 to 41.7% in 2020, and it has been projected that the rate will keep decreasing till 2025 (Table 3).

There is currently no known available data on the global epidemiological trend of unprotected oral sex (a major route of HPV transmission) [35, 39, 40, 41]; however, several countries have recorded a significant increase in the prevalence trend of oral sex [51, 52]. Pertinently, some recent empirical evidence (published between 2017 and 2023) (Table 4) and anecdotal evidence on the prevalence rates of oral sex amongst adolescents and adults in many countries are currently high, and these rates commonly exceed the commonly reported prevalence rates of tobacco use and alcohol use in studies conducted in those same countries [49, 50, 51, 52].

**TABLE 4:** Examples of recently reported prevalence of oral sex among different populations.

Country/Population	Sample	Prevalence of oral sex (%)
Britain [51]	Heterosexuals aged 16–24 years	63.8–74.6
Nigeria [53]	Nurses	49.5
Nigeria [54]	Clinicians (medical doctors, dentists, clinical pharmacists and nurses/midwives)	55.5
Nigeria [55]	Secondary (high) school students	11.8
United States [56]	Heterosexuals aged 15–44 years	>75.0
South Africa [57]	Pregnant and postpartum women	99.7
China [58]	Female sex workers	21.9

Due to the high prevalence of oral sex practices in different parts of the world, it is logical to conclude that the global cases of oral HPV infection will continue to increase [14, 23, 40, 41, 42, 43, 44, 45, 46, 47, 48, 49, 50, 51, 52].

Overall, the above‐described data on the trajectory of the three major oral cancer risk factors – tobacco use, alcohol use and oral sex – depicts that, within the past decades, there has been an ongoing global transition in the epidemiological pattern of oral cancer risk factors [59]. As the world is experiencing a persistent decline in the prevalence of tobacco use and alcohol use, there has been a drastic increase in the prevalence of oral sex in different parts of the world [49, 50, 51, 52]. This paradigm shift occurred most probably because the global public health machinery has over‐concentrated on the control of tobacco and harmful alcohol use, whereas only little attention has been given to safer oral sex promotion and oral HPV infection prevention. Unfortunately, this unequal attention has weakened the impact of global public health efforts against oral cancer. To strengthen the public health efforts for the global eradication of oral cancer, there is a need to focus more on the prevention and control of HPV‐associated oral cancer. To achieve this, it is very paramount to identify the contemporary challenges impeding the global eradication of HPV‐associated oral cancer as such information will be very insightful and instrumental to its eradication efforts.

## CHALLENGES IN HPV‐ASSOCIATED ORAL CANCER PREVENTION AND CONTROL

The challenges facing the world in HPV‐associated oral cancer control are numerous. These challenges range from clinical to social and public health policy issues. However, the major issues are discussed below.

### Lack of validated, inexpensive oral HPV screening and diagnostic tools

Screening tests are designed to identify a disease likely to occur in asymptomatic persons, it achieves this by highlighting risk markers with a high probability of resulting in the disease being screened for [60]. As most oral cancers are preceded by an identifiable premalignant phase, it is reasonable to carry out screening tests for populations at risk of developing oral cancer [60] thereby aiding early diagnosis and prompt management of the same. The commonest test employed in the screening for oral cancer is a systemic oral examination which involves a systematic visual inspection and palpation of the oral cavity under bright light aimed at detecting abnormalities that point towards oral cancer [60]. Specific methods employed for oral HPV screening include oral rinse [61] and oral swabs [61]. Other methods of screening for oral cancer that have been considered by researchers include optical‐based adjuncts, vital staining, cytopathologic platforms, tissue autofluorescence devices and vital rinsing with toluidine blue. However, none of these methods of screening for HPV‐induced oral cancers have been validated in very large trials [60]. Methods that use pictures captured on mobile devices paired with artificial intelligence models, saliva and serum markers sound promising now and may become mainstream in the future.

Though most HPV infections follow a subclinical course lasting between 12 and 24 months before resolution [61, 62], screening for oral HPV can contribute to early identification of the population at risk of developing HPV‐associated oral cancer.

It is also notable that HPV serology and HPV viral culture are unreliable in the diagnosis of HPV as the virus cannot be cultured and most people infected by HPV do not form antibodies against it, the diagnostic value of antibodies found in saliva is unknown [63]. However, the diagnosis of oral HPV infections can be made using light microscopy of biopsied samples, viral antigens detection, viral nucleic acid detection (such as in situ hybridisation, e.g. RNAScope, polymerase chain reaction) and immunohistochemistry [63].

The above‐identified oral HPV screening and diagnostic approaches are very laudable and state‐of‐the‐art; however, their application is largely underutilised in several countries of the world, particularly the LMICs, due to the scarcity of resources and limited technical know‐how [64, 65].

### Global public illiteracy on oral HPV infection

The ability to make informed health decisions concerning risk factors, prevention and illness behaviour constitutes oral health literacy with important indicators including finding, understanding, appraising, judging and deciding or applying relevant information. Different studies from different parts of the world had been conducted on health literacy on oral HPV infection and oral cancer; unfortunately, many of these studies found that such literacy level was very low among the general populations [66, 67, 68, 69]. For example, among college students in the USA – a group of highly enlightened individuals – assessed for oral health literacy related to HPV and oral cancers, low health literacy accounted for their misconceptions about HPV and inadequate HPV vaccine uptake [66, 67, 69]. Similarly, in a very recent multi‐country online survey of 886 dental students – prospective oral health experts – in six LMICs (Egypt, India, Pakistan, Sudan, Saudi Arabia and United Arab Emirate) about the knowledge about HPV and oral cancer, it was found that not many of them understood the association between HPV and oral cancer [70]. Obviously, this shows the need for massive public health literacy development programmes on HPV‐associated OC across the world [71].

Health literacy is a significant determinant of health‐related behaviour – it is about knowledge that could trigger preventive actions. Clinicians, particularly oral health professionals, have significant roles to play in increasing health literacy on oral HPV infection and oral cancer [72, 73]. Unfortunately, several clinicians, including dentists, lack adequate knowledge of the protective measures to prevent the transmission of HPV through oral sex – the major route of oral HPV infection [54, 72]. Hence, by improving clinicians' knowledge on oral HPV infection and oral cancer, their oral HPV infection‐related and oral cancer prevention practices will improve. Moreover, this will ultimately enhance their willingness/enthusiasm to engage in patient education on oral HPV infection and HPV‐associated OC [54, 72].

Vaccination against HPV is very effective in the prevention of HPV‐induced cancers, including oral cancer [74]. However, high HPV vaccine hesitancy and low HPV vaccine uptake have been recorded in several countries due to low awareness, inadequate knowledge and misconceptions concerning HPV and oral cancer [75, 76, 77, 78]. To improve the knowledge of HPV and its associated risks, several interventions including school‐based interventions had been implemented in several parts of the world [71, 79]. However, an overwhelming majority (>90%) of community‐based HPV in several countries were not focused on oral HPV infection and HPV‐induced oral cancer, rather they were focused on non‐oral HPV infections and non‐oral cancers [71, 79, 80].

Due to the high prevalence rates of risky sexual behaviours among them, compared to other age groups, the youths – adolescents and young adults – are the most vulnerable population group when it comes to HPV infection [51, 52]. Hence, school‐based interventions are very influential tools that can be used to reach this population group [79]. The rationale is that school‐age youths constitute an important group for HPV education to communicate risk factors and preventive strategies [79]. School‐based health interventions could reach this group and convey important messages which could make a difference, especially to ensure safer oral sex practices and acceptability of oral screening and HPV vaccination [71, 79, 80].

Historically, the public knowledge of HIV/AIDS has been increasing rapidly, whereas the knowledge of HPV and associated cancers has been considerably low [81]. In Europe, HPV knowledge is still modest but varies across member European countries [82]. There is limited information about HPV in the context of sexually transmitted infections and cancer [77], and more importantly concerning oral HPV infection which is mostly acquired through oral sex [83]. In general, there is a need for more implementation research concerning risk communication and preventive behaviour across the world [83, 84]. Researchers have noted the need to promote HPV knowledge and related cancer risks through education programmes [71, 79, 85, 86]. Such education programmes should also promote the knowledge of HPV vaccine and its safety to enhance acceptability and coverage [79, 86, 87, 88].

### Sociocultural factors and oral HPV infection

Sociocultural factors are very crucial to human health [89]. Such factors, including normative beliefs, cultural norms and religion, affect HPV vaccine acceptability, especially in the low‐income countries [90]. For example, in some Asian countries, some Muslims think that vaccines are not halal (i.e. not permissible under Islam). It has been noted that when the HPV vaccine was approved for use, many Islamic experts raised concerns about halal uncertainty [90]. The only positive argument is that the notion of halal is not absolute in Islam because an impure substance is acceptable to preserve lives when there are no alternatives. Islam shapes sexual behaviour and related health and illness behaviour in most Muslim‐dominated Middle East and North Africa [91]. Although oral sex is permissible in Islam, many Muslims assume that seeking protection against sexually transmitted diseases is due to indiscriminate sex, which is not permitted in Islam and many other religions. Religious taboos or beliefs have also been reported as the crucial reasons for vaccination hesitancy among adherents of Sikhism and Hinduism too [92]. The implication is that religious beliefs could encourage vaccine hesitancy. Hence, it is recommended that religion‐based strategies and interventions that will encourage HPV vaccine uptakes should be considered. Pertinently, this can be achieved through the involvement of religious leaders in the development, implementation and evaluation of such strategies and interventions.

In most LMICs, there is a culture of silence and shyness concerning sexuality issues [93]. Therefore, discussing oral HPV infection, especially through oral sex might not be welcome in many sociocultural contexts. There are also gender issues in HPV infection and vaccination because of the feminisation and moralisation of the process within the patriarchal context [94]. It is as if HPV is another disease for the morally infirmed and females [95]. Such conception imposes an undue moral burden of HPV and related‐health behaviour of women [94].

Based on the above, a culture‐sensitive approach in oral HPV infection control should be encouraged. A twofold strategy should be adopted for this approach. First, the decision‐makers should be careful not to reinforce a culture of inequality which suppresses or undermines certain groups. Second, it is important to take cognise of aspects that will help to ensure the acceptability of the intervention. For instance, culture‐sensitive communication is crucial to enhance intervention acceptability and convey appropriate messages.

### Weak global research capacity on oral HPV infection and HPV‐associated oral cancer control

Health workforce is often in short supply – this has always been a fundamental problem affecting several countries, especially the LMICs. Therefore, meeting the need for certain specialists is a significant challenge in these countries. For instance, a comprehensive and effective health system is essential to curb various forms of preventable morbidities and mortalities. Unfortunately, there is always a critical shortage of oral health specialists to manage the high burden of oral diseases in most LMICs [96]. Such shortage hampers the supply and delivery of adequate oral health services. The problem of a shortage of specialists is also compounded by weak research capacity.

Overall, the global research capacity on oral cancer, particularly on HPV‐associated oral cancer, is currently weak. For instance, it has been observed that HPV research productivity in Africa is very low due to considerably low research funding within the continent [97]. The research agenda is not only about the causative mechanism of HPV‐cancer relations but also about vaccine uptake which is considerably low even in Western countries [98]. Basic intervention research is required to improve the knowledge of oral HPV infection and promote the uptake of the HPV vaccine worldwide. With the rise in the global burden of HPV‐induced oral cancers, there is also the imminent need to intensify research to unravel the factors that control the progression from oral HPV infection to oral cancer [98].

### Absence of policies on national preventive programmes on HPV‐associated oral cancer

National cancer screening and vaccination programmes are the two major cancer cost‐effective preventive programmes commonly adopted in different countries of the world [99, 100, 101, 102]. National screening programmes for prostate, cervical and breast cancer are the most common cancer screening programmes worldwide [101, 103, 104]. Unfortunately, policies on national screening programme on oral cancer are non‐existence in virtually all countries of the world [60]. This is an issue of serious concern, considering the rising burden of oral cancer, including HPV‐associated oral cancer, recorded globally [9, 10, 14, 20, 21, 22]. Hence, it is recommended that such programmes are provided in all countries of the world.

Vaccination against HPV also goes a long way in the global eradication of HPV‐associated oral cancer [26, 105, 106, 107]. The introduction of HPV vaccination into the national vaccination programmes, for example in Britain and United States of America, has brought about a decrease in the overall burden of HPV‐associated diseases in both countries [105, 108]. However, as of June 2020, only 55% (107/194) of the WHO member states have introduced HPV vaccination into their national immunisation programmes [26]. Only in Europe and the Americas, the majority (Europe – 77%; Americas – 85%) of their countries have such vaccination programmes in place; however, such is not the case for other continents, especially Africa, where about half or more of their countries lack such programmes [26]. So far, only 6% (5/194) of the WHO member states – all of which are rich countries – have achieved 90% coverage of full dose HPV vaccination across its populations [26]. This shows that an overwhelming majority of the world population are not vaccinated or yet to be fully vaccinated against HPV. To achieve herd immunity against HPV, it is recommended that all countries of the world should prioritise on the formulation and implementation of effective policies geared towards the introduction and massive roll out of HPV vaccination into their national vaccination programmes.

## LIMITATIONS OF THIS STUDY

Being a narrative review, this study has its limitations. First, the review relied only on secondary sources of data on oral cancers, of which they are very limited as there are relatively low volumes of empirical research outputs on oral cancer, especially HPV‐associated oral cancers [97]. Second, considering the objectives and broad scope of this review, it is not feasible to conduct it using a structured methodological design (i.e. a systematic or scoping review design), as such designs are ideal for highly focused research questions [109].

Regardless of the above limitations, this narrative review has its own strengths. This review is believed to provide a comprehensive overview of contemporary evidence on the global health trends of HPV‐associated oral cancer. Second, this review has provided deeper and broader insights on the key areas of oral cancer eradication that requires global and urgent public health attention.

## CONCLUSION

The burden of oral cancer, especially HPV‐associated oral cancers, has increased over the years, in spite of the global efforts channelled towards global cancer control. Oral cancer is caused by three major risk factors, which are tobacco use, alcohol consumption and oral HPV infection. There has been a global transition in the epidemiological pattern of oral cancer these three major risk factors; the global prevalence of tobacco and alcohol use is declining, whereas the prevalence of oral sex keeps increasing across different populations. This review critically discussed the increasing global burden of HPV‐associated oral cancer – which is a major contributor to the rising global oral cancer burden – as well as the challenges associated with the global eradication of such oral cancer. These challenges include the underutilisation of relevant screening and diagnostic practices, low level of health illiteracy, unhelpful sociocultural factors, absence of policies on national preventive programmes and weak global research capacity on oral HPV infection and HPV‐associated oral cancer. Apart from the need for improved policies and research capacity in this area, there is a need for more implementation research concerning risk communication and preventive behaviour across the world concerning oral HPV infection.

## AUTHOR CONTRIBUTIONS

*Conceptualisation; data curation; formal analysis; funding acquisition; investigation; methodology; project administration; resources; software; supervision; validation; visualisation; writing – original draft; writing – review and editing*: Kehinde Kazeem Kanmodi. *Investigation; methodology; resources; writing – original draft; writing – review and editing*: Jimoh Amzat. *Investigation; methodology; resources; writing – original draft; writing – review and editing*: Jacob Njideka Nwafor. *Resources*: Lawrence Achilles Nnyanzi.

## CONFLICT OF INTEREST STATEMENT

The authors have no conflicts of interest to declare.

## FUNDING INFORMATION

This study was self‐funded.

## ETHICAL STATEMENT

Not applicable. This study did not collect data from human or animal subjects but from an open research repository.

## Data Availability

Data sharing is not applicable to this article as no new data were created or analysed in this study.

## References

[1] Pestle W , Colvard M . A disease without history? Evidence for the antiquity of head and neck cancers. In: Radosevich J.A. , editor. Head & Neck Cancer: Current Perspectives, Advances, and Challenges. Springer; 2013:5‐36.

[2] Cervino G . Milestones of dentistry: advent of anesthetics in oral surgery. Dent J (Basel). 2019;7(4):112. doi:10.3390/dj7040112 31835643 PMC6960780

[3] Ebbell B . The Papyrus Ebers: The Greatest Egyptian Medical Document. Levin & Munksgaard; 1937;1‐135.

[4] Inchingolo F , Santacroce L , Ballini A , et al. Oral cancer: a historical review. Int J Environ Res Public Health. 2020;17(9):3168. doi:10.3390/ijerph17093168 32370133 PMC7246763

[5] National Institute of Dental and Craniofacial Research . Oral cancer [Internet]. National Institute of Dental and Craniofacial Research; 2023. Accessed 19 July 2023. https://www.nidcr.nih.gov/health‐info/oral‐cancer

[6] GBD 2017 Disease and Injury Incidence and Prevalence Collaborators . Global, regional, and national incidence, prevalence, and years lived with disability for 354 diseases and injuries for 195 countries and territories, 1990–2017: a systematic analysis for the global burden of disease study 2017. Lancet. 2018;392(10159):1789‐1858. doi:10.1016/S0140-6736(18)32279-7 30496104 PMC6227754

[7] WHO (World Health Organization) . Global status report on alcohol and health. WHO (World Health Organization); 2018. Accessed 16 January 2022. https://apps.who.int/iris/rest/bitstreams/1151838/retrieve

[8] Ferlay J , Colombet M , Soerjomataram I , et al. Estimating the global cancer incidence and mortality in 2018: GLOBOCAN sources and methods. Int J Cancer. 2019;144(8):1941‐1953. doi:10.1002/ijc.31937 30350310

[9] Ren ZH , Hu CY , He HR , Li YJ , Lyu J . Global and regional burdens of oral cancer from 1990 to 2017: results from the global burden of disease study. Cancer Commun (Lond). 2020;40(2‐3):81‐92. doi:10.1002/cac2.12009 32067418 PMC7163731

[10] Du M , Nair R , Jamieson L , Liu Z , Bi P . Incidence trends of lip, oral cavity, and pharyngeal cancers: global burden of disease 1990–2017. J Dent Res. 2020;99(2):143‐151. doi:10.1177/0022034519894963 31874128

[11] Kujan O , Farah CS , Johnson NW . Oral and oropharyngeal cancer in the Middle East and North Africa: incidence, mortality, trends, and gaps in public databases as presented to the Global Oral Cancer Forum. Transl Res in Oral Oncology. 2017;2:1‐9. 2057178×17698480.

[12] Sung H , Ferlay J , Siegel RL , Global cancer statistics 2020: GLOBOCAN estimates of incidence and mortality worldwide for 36 cancers in 185 countries. CA Cancer J Clin. 2021;71(3):209‐249. doi:10.3322/caac.21660 33538338

[13] Torre LA , Bray F , Siegel RL , Ferlay J , Lortet‐Tieulent J , Jemal A . Global cancer statistics, 2012. CA Cancer J Clin. 2015;65(2):87‐108. doi:10.3322/caac.21262 25651787

[14] Conway DI , Purkayastha M , Chestnutt IG . The changing epidemiology of oral cancer: definitions, trends, and risk factors. Br Dent J. 2018;9225(9):867‐873. doi:10.1038/sj.bdj.2018.922 30412558

[15] Harris JP , Iturriza‐Gomara M , O'Brien SJ . Estimating disability‐adjusted life years (DALYs) in community cases of norovirus in England. Viruses. 2019;11(2):184. doi:10.3390/v11020184 30795617 PMC6410119

[16] Ribeiro‐Rotta RF , Rosa EA , Milani V . The cost of oral cancer: a systematic review. PLoS One. 2022;17(4):e0266346. doi:10.1371/journal.pone.0266346 35446870 PMC9022815

[17] Martins AM , Barreto SM , dos Santos‐Neto PE , et al. Greater access to information on how to prevent oral cancer among elderly using primary health care. Cien Saude Colet. 2015;20(7):2239‐2253 (English, Portuguese). doi:10.1590/1413-81232015207.15272014 26132263

[18] Nieminen M , Atula T , Bäck L , Mäkitie A , Jouhi L , Aro K . Factors influencing patient and health care delays in oropharyngeal cancer. J Otolaryngol Head Neck Surg. 2020;49(1):22. doi:10.1186/s40463-020-00413-w 32326977 PMC7181590

[19] Kanmodi KK , Amzat Jimoh , Nnyanzi LA . The state of global research on poverty after adoption of sustainable development goals. Public Health Chall. 2023;2(3):e110. 10.1002/puh2.110 PMC12039691 40496291

[20] Forman D , de Martel C , Lacey CJ , et al. Global burden of human papillomavirus and related diseases. Vaccine. 2012;30(5):F12‐F23. doi:10.1016/j.vaccine.2012.07.055 23199955

[21] de Martel C , Plummer M , Vignat J , Franceschi S . Worldwide burden of cancer attributable to HPV by site, country and HPV type. Int J Cancer. 2017;141(4):664‐670. doi:10.1002/ijc.30716 28369882 PMC5520228

[22] Lechner M , Liu J , Masterson L , Fenton TR . HPV‐associated oropharyngeal cancer: epidemiology, molecular biology and clinical management. Nat Rev Clin Oncol. 2022;19(5):306‐327. doi:10.1038/s41571-022-00603-7 35105976 PMC8805140

[23] Gillison ML , Chaturvedi AK , Anderson WF , Fakhry C . Epidemiology of human papillomavirus‐positive head and neck squamous cell carcinoma. J Clin Oncol. 2015;33(29):3235‐3242. doi:10.1200/JCO.2015.61.6995 26351338 PMC4979086

[24] Schiffman M , Doorbar J , Wentzensen N , et al. Carcinogenic human papillomavirus infection. Nat Rev Dis Primers. 2016;2:16086. doi:10.1038/nrdp.2016.86 27905473

[25] Petrosky E , Bocchini JA Jr , Hariri S , et al.; Centers for Disease Control and Prevention (CDC) . Use of 9‐valent human papillomavirus (HPV) vaccine: updated HPV vaccination recommendations of the advisory committee on immunization practices. MMWR Morb Mortal Wkly Rep. 2015;64(11):300‐304. 25811679 PMC4584883

[26] Bruni L , Saura‐Lázaro A , Montoliu A , et al. HPV vaccination introduction worldwide and WHO and UNICEF estimates of national HPV immunization coverage 2010–2019. Prev Med. 2021;144:106399. doi:10.1016/j.ypmed.2020.106399 33388322

[27] Serrano B , Brotons M , Bosch FX , Bruni L . Epidemiology and burden of HPV‐related disease. Best Pract Res Clin Obstet Gynaecol. 2018;47:14‐26. doi:10.1016/j.bpobgyn.2017.08.006 29037457

[28] Hwang TZ , Hsiao JR , Tsai CR , Chang JS . Incidence trends of human papillomavirus‐related head and neck cancer in Taiwan, 1995–2009. Int J Cancer. 2015;137(2):395‐408. doi:10.1002/ijc.29330 25395239

[29] Attner P , Du J , Näsman A , et al. The role of human papillomavirus in the increased incidence of base of tongue cancer. Int J Cancer. 2010;126(12):2879‐2884. doi:10.1002/ijc.24994 19856308

[30] Faraji F , Rettig EM , Tsai HL , et al. The prevalence of human papillomavirus in oropharyngeal cancer is increasing regardless of sex or race, and the influence of sex and race on survival is modified by human papillomavirus tumor status. Cancer. 2019;125(5):761‐769. doi:10.1002/cncr.31841 30521092

[31] Wittekindt C , Wagner S , Bushnak A , et al. Increasing incidence rates of oropharyngeal squamous cell carcinoma in Germany and significance of disease burden attributed to human papillomavirus. Cancer Prev Res (Phila). 2019;12(6):375‐382. doi:10.1158/1940-6207.CAPR-19-0098 31003993

[32] Zamani M , Grønhøj C , Jensen DH , et al. The current epidemic of HPV‐associated oropharyngeal cancer: an 18‐year Danish population‐based study with 2,169 patients. Eur J Cancer. 2020;134:52‐59. doi:10.1016/j.ejca.2020.04.027 32460181

[33] Girardi FM , Wagner VP , Martins MD , Abentroth AL , Hauth LA . Prevalence of p16 expression in oropharyngeal squamous cell carcinoma in southern Brazil. Oral Surg Oral Med Oral Pathol Oral Radiol. 2020;130(6):681‐691. doi:10.1016/j.oooo.2020.08.021 32981865

[34] Donà MG , Rollo F , Pichi B , et al. Evolving profile of HPV‐driven oropharyngeal squamous cell carcinoma in a national cancer institute in Italy: a 10‐year retrospective study. Microorganisms. 2020;8(10):1498. doi:10.3390/microorganisms8101498 33003378 PMC7599861

[35] Kumar M , Nanavati R , Modi TG , Dobariya C . Oral cancer: etiology and risk factors: a review. J Cancer Res Ther. 2016;12(2):458‐463. doi:10.4103/0973-1482.186696 27461593

[36] Shaw R , Beasley N . Aetiology and risk factors for head and neck cancer: United Kingdom National Multidisciplinary Guidelines. J Laryngol Otol. 2016;130(S2):S9‐S12. doi:10.1017/S0022215116000360 27841107 PMC4873944

[37] Niaz K , Maqbool F , Khan F , Bahadar H , Ismail Hassan F , Abdollahi M . Smokeless tobacco (paan and gutkha) consumption, prevalence, and contribution to oral cancer. Epidemiol Health. 2017;39:e2017009. doi:10.4178/epih.e2017009 28292008 PMC5543298

[38] Barul C , Carton M , Radoï L , et al. ICARE study group. Occupational exposure to petroleum‐based and oxygenated solvents and oral and oropharyngeal cancer risk in men: a population‐based case‐control study in France. Cancer Epidemiol. 2019;59:22‐28. doi:10.1016/j.canep.2019.01.005 30658217

[39] Kreimer AR , Clifford GM , Boyle P , Franceschi S . Human papillomavirus types in head and neck squamous cell carcinomas worldwide: a systematic review. Cancer Epidemiol Biomarkers Prev. 2005;14(2):467‐475. doi:10.1158/1055-9965.EPI-04-0551 15734974

[40] Pantanowitz L , Khammissa RA , Lemmer J , Feller L . Oral HIV‐associated Kaposi sarcoma. J Oral Pathol Med. 2013;42(3):201‐207. doi:10.1111/j.1600-0714.2012.01180.x 22672182

[41] Katsanos KH , Roda G , Brygo A , Delaporte E , Colombel JF . Oral cancer and oral precancerous lesions in inflammatory bowel diseases: a systematic review. J Crohns Colitis. 2015;9(11):1043‐1052. doi:10.1093/ecco-jcc/jjv122 26163301

[42] Yakin M , Gavidi RO , Cox B , Rich A . Oral cancer risk factors in New Zealand. N Z Med J. 2017;130(1451):30‐38. 28253242

[43] Negri E , Franceschi S , Bosetti C , et al. Selected micronutrients and oral and pharyngeal cancer. Int J Cancer. 2000;86(1):122‐127. doi:10.1002/(sici)1097-0215(20000401)86 10728605

[44] Garavello W , Lucenteforte E , Bosetti C , La Vecchia C . The role of foods and nutrients on oral and pharyngeal cancer risk. Minerva Stomatol. 2009;58(1‐2):25‐34. 19234434

[45] Lawal AO , Kolude B , Adeyemi BF , Lawoyin JO , Akang EE . Serum antioxidant vitamins and the risk of oral cancer in patients seen at a tertiary institution in Nigeria. Niger J Clin Pract. 2012;15(1):30‐33. doi:10.4103/1119-3077.94093 22437085

[46] Akhiwu BI , Akhiwu HO , Afolaranmi T , et al. Characterization of high risk human papilloma virus genotypes associated with oropharyngeal cancers in a Nigerian population. Pan Afr Med J. 2021;38:40. doi:10.11604/pamj.2021.38.40.27309 33777308 PMC7955602

[47] Dhull AK , Atri R , Dhankhar R , Chauhan AK , Kaushal V . Major risk factors in head and neck cancer: a retrospective analysis of 12‐year experiences. World J Oncol. 2018;9(3):80‐84. doi:10.14740/wjon1104w 29988794 PMC6031231

[48] Yete S , D'Souza W , Saranath D . High‐risk human papillomavirus in oral cancer: clinical implications. Oncology. 2018;94(3):133‐141. doi:10.1159/000485322 29241220

[49] WHO (World Health Organization) . WHO Global Report on Trends in Prevalence of Tobacco use 2000–2025. 3rd ed. World Health Organization; 2019. Accessed 16 January 2022. https://apps.who.int/iris/rest/bitstreams/1263754/retrieve

[50] Varela‐Centelles P, Seoane J, Ulloa‐Morales Y, Estany‐Gestal A, Blanco‐Hortas A, García‐Pola MJ, Seoane‐Romero JM. Oral cancer awareness in North‐Western Spain: a population‐based study. Med Oral Patol Oral Cir Bucal. 2021;26(4):e518‐e525. doi:10.4317/medoral.24401 34162825 PMC8254879

[51] Lewis R , Tanton C , Mercer CH , et al. Heterosexual practices among young people in Britain: evidence from three national surveys of sexual attitudes and lifestyles. J Adolesc Health. 2017;61(6):694‐702. doi:10.1016/j.jadohealth.2017.07.004 29169520 PMC5723633

[52] Morhason‐Bello IO , Kabakama S , Baisley K , Francis SC , Watson‐Jones D . Reported oral and anal sex among adolescents and adults reporting heterosexual sex in sub‐Saharan Africa: a systematic review. Reprod Health. 2019;16(1):48. doi:10.1186/s12978-019-0722-9 31060573 PMC6501425

[53] Kanmodi KK , Nwafor JN , Amoo BA , Nnyanzi LA , Ogbeide ME , Hundeji AA . Knowledge of the health implications of oral sex among registered nurses in Nigeria: an online pilot study. J Health Allied Sci. 2023;13(1):46‐52.

[54] Kanmodi KK , Nwafor JN , Eze UA , et al. Factors determining the willingness of Nigerian clinicians to recommend protected oral sex: an online exploratory study. Oral. 2022;2(4):299‐315.

[55] Kanmodi K , Fagbule O , Ogunniyi K , et al. Determinants of sexual practices among secondary school students in Nigeria: focusing on socio‐cultural and school‐related factors. Rwanda Med J. 2020;77(4):32‐37.

[56] Habel MA , Leichliter JS , Dittus PJ , Spicknall IH , Aral SO . Heterosexual anal and oral sex in adolescents and adults in the United States, 2011–2015. Sex Transm Dis. 2018;45(12):775‐782. doi:10.1097/OLQ.0000000000000889 29965947 PMC6753934

[57] Joseph Davey D, Farley E , Gomba Y , Coates T , Myer L . Sexual risk during pregnancy and postpartum periods among HIV‐infected and ‐uninfected South African women: implications for primary and secondary HIV prevention interventions. PLoS One. 2018;13(3):e0192982. doi:10.1371/journal.pone.0192982 29509759 PMC5839542

[58] Zhou X , Ma Q , Pan X , Chen L , Wang H , Jiang T . The prevalence and correlates of oral sex among low‐tier female sex workers in Zhejiang province, China. PLoS One. 2020;15(9):e0238822. doi:10.1371/journal.pone.0238822 32898155 PMC7478619

[59] Rettig EM , D'Souza G . Epidemiology of head and neck cancer. Surg Oncol Clin N Am. 2015;24(3):379‐396. doi:10.1016/j.soc.2015.03.001 25979389

[60] Warnakulasuriya S , Kerr AR . Oral cancer screening: past, present, and future. J Dent Res. 2021;100(12):1313‐1320. doi:10.1177/00220345211014795 34036828 PMC8529297

[61] Gipson BJ , Robbins HA , Fakhry C , D'Souza G . Sensitivity and specificity of oral HPV detection for HPV‐positive head and neck cancer. Oral Oncol. 2018;77:52‐56. doi:10.1016/j.oraloncology.2017.12.008 29362127 PMC5788034

[62] Betz SJ . HPV‐related papillary lesions of the oral mucosa: a review. Head Neck Pathol. 2019;13(1):80‐90. doi:10.1007/s12105-019-01003-7 30693456 PMC6405797

[63] Syrjänen S . Oral manifestations of human papillomavirus infections. Eur J Oral Sci. 2018;126(1):49‐66. doi:10.1111/eos.12538 30178562 PMC6174935

[64] Tax CL , Haslam SK , Brillant M , Doucette HJ , Cameron JE , Wade SE . Oral cancer screening: knowledge is not enough. Int J Dent Hyg. 2017;15(3):179‐186. doi:10.1111/idh.12172 26333090

[65] Nieves‐Rivera A , Benchetrit L , Kan K , Tucker S , Johnson M , Edwards H . Use of tongue base palpation among oral healthcare providers: cross‐sectional survey. Am J Otolaryngol. 2023;44(2):103765. doi:10.1016/j.amjoto.2022.103765 36603380

[66] Albright AE , Allen RS . HPV misconceptions among college students: the role of health literacy. J Community Health. 2018;43(6):1192‐1200. doi:10.1007/s10900-018-0539-4 29922992

[67] Monroy E , Griner, S . Oral health literacy and HPV: an unrecognized risk for college students. UNTHSC Scholar. Accessed 06 March 2023. https://hdl.handle.net/20.500.12503/30195

[68] Kanmodi K , Kanmodi P , Ogbeide M , Nwafor J . Head and neck cancer literacy in Nigeria: a systematic review of the literature. Annals of Public Health Issues. 2021;1(1):25‐49. doi:10.2478/aphi-2021-0004

[69] El Mansouri N , Ferrera L , Kharbach A , et al. Awareness and knowledge associated to human papillomavirus infection among university students in Morocco: a cross‐sectional study. PLoS One. 2022;17(7):e0271222. doi:10.1371/journal.pone.0271222 35802731 PMC9269923

[70] Lingam AS , Koppolu P , Alhussein SA , et al. Dental students' perception, awareness and knowledge about HPV infection, vaccine, and its association with oral cancer: a multinational study. Infect Drug Resist. 2022;15:3711‐3724. doi:10.2147/IDR.S365715 35855757 PMC9288190

[71] Kanmodi KK , Fagbule OF . Towards head and neck cancer prevention in Nigeria: insights from the CHANCE programme. Head Neck. 2020;2:16.

[72] Vázquez‐Otero C , Vamos CA , Thompson EL , et al. Assessing dentists' human papillomavirus‐related health literacy for oropharyngeal cancer prevention. J Am Dent Assoc. 2018;149(1):9‐17. doi:10.1016/j.adaj.2017.08.021 29031503 PMC5756503

[73] Salami AA , Kanmodi KK , Nnyanzi LA . Re‐emphasizing the roles of general medical and dental practitioners regarding oral cancer eradication in Nigeria. Acta Medica Martiniana. 2021;21(3):90‐102. doi:10.2478/acm-2021-0012

[74] Athanasiou A , Bowden S , Paraskevaidi M , et al. HPV vaccination and cancer prevention. Best Pract Res Clin Obstet Gynaecol. 2020;65:109‐124. doi:10.1016/j.bpobgyn.2020.02.009 32284298

[75] Patel PR , Berenson AB . Sources of HPV vaccine hesitancy in parents. Hum Vaccin Immunother. 2013;9(12):2649‐2653. doi:10.4161/hv.26224 23982270 PMC4162068

[76] Kops NL , Hohenberger GF , Bessel M , et al. Knowledge about HPV and vaccination among young adult men and women: results of a national survey. Papillomavirus Res. 2019;7:123‐128. doi:10.1016/j.pvr.2019.03.003 30885798 PMC6426699

[77] Netfa F , Tashani M , Booy R , King C , Rashid H , Skinner SR . Knowledge, attitudes and perceptions of immigrant parents towards human papillomavirus (HPV) vaccination: a systematic review. Trop Med Infect Dis. 2020;5(2):58. doi:10.3390/tropicalmed5020058 32283644 PMC7344469

[78] Szilagyi PG , Albertin CS , Gurfinkel D , et al. Prevalence and characteristics of HPV vaccine hesitancy among parents of adolescents across the US. Vaccine. 2020;38(38):6027‐6037. doi:10.1016/j.vaccine.2020.06.074 32758380 PMC9495911

[79] Amzat J , Kanmodi KK , Aminu K , Egbedina EA . School‐based interventions on human papillomavirus in Africa: a systematic scoping review. Venereology. 2023;2(1):43‐58. doi:10.3390/venereology2010004

[80] Kanmodi KK , Fagbule OF . Does head and neck cancer (HNC) education have impact on adolescents' knowledge and attitudes towards HNC and HNC peer‐education? An example from Nigeria. Int J Child Adolesc Health. 2018;11(3):343‐347.

[81] Hendry M , Lewis R , Clements A , Damery S , Wilkinson C . “HPV? Never heard of it!”: a systematic review of girls' and parents’ information needs, views and preferences about human papillomavirus vaccination. Vaccine. 2013;31(45):5152‐5167. doi:10.1016/j.vaccine.2013.08.091 24029117

[82] López N , Garcés‐Sánchez M , Panizo MB , et al. HPV knowledge and vaccine acceptance among European adolescents and their parents: a systematic literature review. Public Health Rev. 2020;41:10. doi:10.1186/s40985-020-00126-5 32435520 PMC7222509

[83] Taberna M , Mena M , Pavón MA , Alemany L , Gillison ML , Mesía R . Human papillomavirus‐related oropharyngeal cancer. Ann Oncol. 2017;28(10):2386‐2398. doi:10.1093/annonc/mdx304 28633362

[84] Glik DC . Risk communication for public health emergencies. Annu Rev Public Health. 2007;28:33‐54. doi:10.1146/annurev.publhealth.28.021406.144123 17222081

[85] Kanmodi KK , Fagbule OF , Ogbeide ME , et al. Knowledge of senior secondary school students in Nigeria about head and neck cancer: implications on prevention strategies. Malawi Med J. 2022;34(3):236‐243. 10.4314/mmj.v34i3.4 PMC9641603 36406094

[86] Fagbule OF , Kanmodi KK , Aliemeke EO , et al. Knowledge of HPV and HPV vaccines among senior secondary (high) school students in Nigeria: implications on cancer prevention strategies, CHANCE study. Popul Med. 2020;2(October):31.

[87] Kitur H , Horowitz AM , Beck K , Wang MQ . HPV knowledge, vaccine status, and health literacy among university students. J Cancer Educ. 2022;37(6):1606‐1613. doi:10.1007/s13187-021-01997-1 33768470

[88] Zhang E , Dai Z , Wang S , Wang X , Zhang X , Fang Q . Vaccine literacy and vaccination: a systematic review. Int J Public Health. 2023;68:1605606. doi:10.3389/ijph.2023.1605606 36866001 PMC9970990

[89] Amzat J , Razum O . African Culture and Health. In: Towards a Sociology of Health Discourse in Africa. Springer International Publishing; 2018. doi:10.1007/978-3-319-61672-8_5

[90] Wong LP , Wong PF , Megat Hashim MMAA , et al. Multidimensional social and cultural norms influencing HPV vaccine hesitancy in Asia. Hum Vaccin Immunother. 2020;16(7):1611‐1622. doi:10.1080/21645515.2020.1756670 32429731 PMC7482900

[91] Hamdi S . The impact of teachings on sexuality in Islam on HPV vaccine acceptability in the Middle East and North Africa region. J Epidemiol Glob Health. 2018;7(1):S17‐S22. doi:10.1016/j.jegh.2018.02.003 29801588 PMC7386444

[92] Kibongani Volet A, Scavone C , Catalán‐Matamoros D , Capuano A . Vaccine hesitancy among religious groups: reasons underlying this phenomenon and communication strategies to rebuild trust. Front Public Health. 2022;10:824560. doi:10.3389/fpubh.2022.824560 35198525 PMC8858841

[93] Usonwu I , Ahmad R , Curtis‐Tyler K . Parent‐adolescent communication on adolescent sexual and reproductive health in sub‐Saharan Africa: a qualitative review and thematic synthesis. Reprod Health. 2021;18(1):202. doi:10.1186/s12978-021-01246-0 34629082 PMC8504018

[94] Daley EM , Vamos CA , Thompson EL , et al. The feminization of HPV: how science, politics, economics and gender norms shaped U.S. HPV vaccine implementation. Papillomavirus Res. 2017;3:142‐148. doi:10.1016/j.pvr.2017.04.004 28720448 PMC5883212

[95] Siu JY , Fung TKF , Leung LH . Social and cultural construction processes involved in HPV vaccine hesitancy among Chinese women: a qualitative study. Int J Equity Health. 2019;18(1):147. doi:10.1186/s12939-019-1052-9 31533722 PMC6751778

[96] Tiwari R , Bhayat A , Chikte U . Forecasting for the need of dentists and specialists in South Africa until 2030. PLoS One. 2021;16(5):e0251238. doi:10.1371/journal.pone.0251238 33999933 PMC8128226

[97] Kanmodi KK , Egbedina EA , Amzat J , Aminu K , Nnyanzi LA . The state of human papillomavirus research in Africa. Public Health Challenges. 2023;2(1):e72. 10.1002/puh2.72 PMC12039679 40496963

[98] Langsfeld E , Laimins LA . Human papillomaviruses: research priorities for the next decade. Trends Cancer. 2016;2(5):234‐240. doi:10.1016/j.trecan.2016.04.001 27617309 PMC5015772

[99] Huang CC , Lin CN , Chung CH , Hwang JS , Tsai ST , Wang JD . Cost‐effectiveness analysis of the oral cancer screening program in Taiwan. Oral Oncol. 2019;89:59‐65. doi:10.1016/j.oraloncology.2018.12.011 30732960

[100] Thankappan K , Subramanian S , Balasubramanian D , Kuriakose MA , Sankaranarayanan R , Iyer S . Cost‐effectiveness of oral cancer screening approaches by visual examination: systematic review. Head Neck. 2021;43(11):3646‐3661. doi:10.1002/hed.26816 34260118

[101] Sykes K , Tuschick E , Giles EL , Kanmodi KK , Barker J . A protocol to identify the barriers and facilitators for people with severe mental illness and/or learning disabilities for Person centred cancer screening services (PECCS). PLoS One. 2022;17(11):e0278238. doi:10.1371/journal.pone.0278238 36449513 PMC9710752

[102] Zou Z , Fairley CK , Ong JJ , et al. Domestic HPV vaccine price and economic returns for cervical cancer prevention in China: a cost‐effectiveness analysis. Lancet Glob Health. 2020;8(10):e1335‐e1344. doi:10.1016/S2214-109X(20)30277-1 32971056

[103] Sanders LD , Larkins TL , Boyle JN , George SF , Triplett EW , Leypoldt MD . National breast and cervical cancer early detection program partnerships in action. Cancer. 2014;120(16):2612‐2616. doi:10.1002/cncr.28827 25099905

[104] Ilic D , Djulbegovic M , Jung JH , et al. Prostate cancer screening with prostate‐specific antigen (PSA) test: a systematic review and meta‐analysis. BMJ. 2018;362:k3519. doi:10.1136/bmj.k3519 30185521 PMC6283370

[105] Falcaro M , Castañon A , Ndlela B , et al. The effects of the national HPV vaccination programme in England, UK, on cervical cancer and grade 3 cervical intraepithelial neoplasia incidence: a register‐based observational study. Lancet. 2021;398(10316):2084‐2092. doi:10.1016/S0140-6736(21)02178-4 34741816

[106] Kamolratanakul S , Pitisuttithum P . Human papillomavirus vaccine efficacy and effectiveness against cancer. Vaccines (Basel). 2021;9(12):1413. doi:10.3390/vaccines9121413 34960159 PMC8706722

[107] Ellingson MK , Sheikha H , Nyhan K , Oliveira CR , Niccolai LM . Human papillomavirus vaccine effectiveness by age at vaccination: a systematic review. Hum Vaccin Immunother. 2023;19(2):2239085. doi:10.1080/21645515.2023.2239085 37529935 PMC10399474

[108] Liao CI , Francoeur AA , Kapp DS , Caesar MAP , Huh WK , Chan JK . Trends in human papillomavirus‐associated cancers, demographic characteristics, and vaccinations in the US, 2001–2017. JAMA Netw Open. 2022;5(3):e222530. doi:10.1001/jamanetworkopen.2022.2530 35294540 PMC8928005

[109] Munn Z , Peters MDJ , Stern C , Tufanaru C , McArthur A , Aromataris E . Systematic review or scoping review? Guidance for authors when choosing between a systematic or scoping review approach. BMC Med Res Methodol. 2018;18(1):143. doi:10.1186/s12874-018-0611-x 30453902 PMC6245623

